# Identification of Microbial and Proteomic Biomarkers in Early Childhood Caries

**DOI:** 10.1155/2011/196721

**Published:** 2011-10-16

**Authors:** Thomas C. Hart, Patricia M. Corby, Milos Hauskrecht, Ok Hee Ryu, Richard Pelikan, Michal Valko, Maria B. Oliveira, Gerald T. Hoehn, Walter A. Bretz

**Affiliations:** ^1^Department of Periodontics, College of Dentistry, University of Illinois at Chicago, 801 S. Paulina Street, Chicago, IL 60612, USA; ^2^Department of Cariology and Comprehensive Care and Department of Periodontics and Implants, College of Dentistry, New York University, 345 E. 24th Street, New York, NY 10010, USA; ^3^Computer Science Department, Intelligent Systems Program, Department of Biomedical Informatics, University of Pittsburgh Cancer Institute, University of Pittsburgh, Pittsburgh, PA 15232, USA; ^4^Human and Craniofacial Genetics Section, National Institute of Dental and Craniofacial Research, National Institutes of Health, Bethesda, MD 20892, USA; ^5^Department of General Dentistry, UNIMONTES, Montes Claros, MG 39401, Brazil; ^6^Critical Care Medicine Department, Clinical Center, National Institutes of Health (NIH), Bethesda, MD 20892, USA

## Abstract

The purpose of this study was to provide a univariate and multivariate analysis of genomic microbial data and salivary mass-spectrometry proteomic profiles for dental caries outcomes. In order to determine potential useful biomarkers for dental caries, a multivariate classification analysis was employed to build predictive models capable of classifying microbial and salivary sample profiles with generalization performance. We used high-throughput methodologies including multiplexed microbial arrays and SELDI-TOF-MS profiling to characterize the oral flora and salivary proteome in 204 children aged 1–8 years (*n* = 118 caries-free, *n* = 86 caries-active). The population received little dental care and was deemed at high risk for childhood caries. Findings of the study indicate that models incorporating both microbial and proteomic data are superior to models of only microbial or salivary data alone. Comparison of results for the combined and independent data suggests that the combination of proteomic and microbial sources is beneficial for the classification accuracy and that combined data lead to improved predictive models for caries-active and caries-free patients. The best predictive model had a 6% test error, >92% sensitivity, and >95% specificity. These findings suggest that further characterization of the oral microflora and the salivary proteome associated with health and caries may provide clinically useful biomarkers to better predict future caries experience.

## 1. Introduction

Dental caries, the most common disease of childhood, is a complex infectious disease with a multifactorial etiology. The caries process is characterized by interactions between a receptive host and microorganisms with the potential for colonization and pathogenesis. Microbial, genetic, immunological, behavioral, environmental, and socioeconomic factors contribute to risk and determine the occurrence and severity of clinical disease [1, 2]. Of the identified risk factors, the cariogenic oral microbial flora and saliva have received particular research attention. 

Microbiological studies conducted in the past four decades have shown that *Streptococcus mutans* is the chief pathogen associated with childhood dental caries onset and that lactobacilli are associated with dental caries progression [3, 4]. Much of this knowledge has been made possible with the use of traditional culturing methods employing selective media for these pathogens. Recent advances employing microbial molecular techniques have allowed for better understanding of the complexity of the flora associated with oral infections, particularly dental caries. More than 750 oral microbial taxa inhabit the oral cavity [5]. Of those, approximately 50% have yet to be cultivated, and many phyla are yet to be characterized and taxonomically classified. Studies incorporating newer molecular genetic methodologies indicate that a greater diversity of oral microbes are associated with the pathological transition from oral health to caries [6–8]. 

Various salivary constituents, salivary flow rate, and salivary buffering capacity have been correlated with caries risk [9–11]. Saliva is a complex fluid that exercises multiple functions in the oral cavity [12]. Salivary components can play a role in susceptibility and demineralization of the enamel as well as enamel remineralization and resistance to dental caries [11]. While the biological function of most salivary proteins and peptides are not well characterized, many salivary proteins are believed to function in the protection of oral tissues [13, 14]. An array of molecules include mucins, histatins, proline-rich peptides, defensins, lactoferrin, and peroxidases regulate the oral microbial flora by exerting direct antimicrobial effects [10, 13]. In addition, it is likely that there are many as yet to be characterized proteins present in saliva that may be pivotal for protection of oral tissues against microbial, viral, or fungal infections [14]. Whereas most of the functions of saliva have been elucidated through classical biochemical approaches, current proteomic techniques, including high-throughput analysis of the salivary proteome, make it possible to characterize a comprehensive catalogue of all salivary proteins and, possibly, their translational impact on the dynamics of dental caries onset and development [15–17]. 

Schipper et al. [13] demonstrated that surface-enhanced laser desorption/ionization time-of-flight-mass spectrometry (SELDI-TOF-MS) provides a simple and high-throughput method to rapidly identify a large number of differently expressed proteins and peptides in saliva. Although interest in evaluating saliva as a diagnostic fluid for monitoring health is receiving increasing attention [16–20], to date, there have been no robust dental caries studies employing salivary proteome analysis and microbial genomic analysis concomitantly.

To date, the diagnostic utility of assays for individual salivary components or for assays of individual microbes have been of limited clinical utility in assessing risk for childhood caries. Although a chronic disease, the most consistent predictor of caries risk in children remains past caries experience [1]. More effective preventive approaches in dental care require improved methods for the early identification of children at risk for caries. Dental caries may occur secondary to ecologically driven imbalances of oral microbial biofilms. It is conceivable that changes in the salivary proteome may parallel alterations in the microbial flora in caries progression. The purpose of this study is to provide a computational validation framework that permits us to assess the significance of genomic microbial data and salivary mass-spectrometry proteomic profiles for dental caries outcomes. In order to determine potential useful biomarkers for dental caries, a multivariate classification analysis was employed to build predictive models capable of classifying microbial and salivary sample profiles with generalization performance. The study was performed in a high-risk population of young children from an area without fluoridated water, who received minimal professional dental care, representing a natural occurrence of early-onset caries in humans.

## 2. Methods

### 2.1. Demographic Characteristics of Subject Population

The study population consisted of a cohort of children of low socioeconomic urban families who resided in the city of Montes Claros, State of Minas Gerais, Brazil. City water supplies had less-than-optimal fluoride levels of 0.2 ppm, and the population evaluated for the most part (>96%) had not received routine professional dental care [7]. Parents of the children signed consent forms, and four human subjects' institutional review boards approved the study protocol. A total of 204 children, aged 1–8 years old, comprised the study population. Children provided saliva and dental plaque biofilm samples and were subsequently examined for dental caries.

### 2.2. Dental Caries Examination

Two examiners conducted dental caries examinations according to National Institute of Dental and Craniofacial Research criteria [21] modified to distinguish caries lesions with a chalky whitish/yellowish opaque appearance, without clinically detectable loss of substance (white spot lesions), from cavitated carious lesions. Interproximal surface caries were assessed using digital imaging fiber-optic transillumination (DIFOTI, Irvington, NY, USA).

### 2.3. Dental Plaque Biofilm Sampling

Supragingival plaque samples were collected in the morning. Caries-free children had pooled plaque samples collected from three healthy surfaces that may have included anterior and posterior teeth. Caries-active children had plaque samples collected separately from a surface of intact enamel (site 1) and three types of caries lesion: surface of white spot lesions (site 2), surface of initial enamel lesions (site 3), and excavated plaque from deep dentinal lesions (site 4). All caries active subjects provided three to four sites of plaque collected separately from different teeth according to the severity of disease. For intact enamel and white spot lesions, plaque was collected by swiping the tooth surface with a Stimudent (Johnson & Johnson, New York, NY, USA), whereas plaque from cavitated lesions was collected by means of a small Gracey curette (1-2; Hu-Friedy, Chicago, Ill, USA). A total of 448 plaque samples (118 collected from caries-free children and 330 from caries-active children) were used for analysis.

### 2.4. Microbial Genomic Analysis

Isolation of bacterial DNA from samples was performed by employing standard procedures previously described [7]. The reverse-capture checkerboard hybridization assay was used to detect relative levels (abundance) of 82 oral bacterial species or groups. Briefly, reverse-capture DNA probes (complementary oligonucleotide DNAs of known sequence) are used to target polynucleotides of unknown sequence (16S rRNA) bacterial genes in the biological sample solution. Probes were placed on a nylon membrane in separate horizontal lanes using a Mini Slot apparatus. 16S rRNA genes from plaque samples were PCR-amplified using a specific labeled primer. Hybridizations were performed in vertical channels in a Miniblotter apparatus with labeled amplicons (target 16S rRNA genes) for up to 45 samples. A total of 1,350 hybridizations were performed simultaneously using a single membrane. Standard chemifluorescence detection was performed using the Storm Imaging System (Amersham, Piscataway, NJ, USA). For each spot on the membranes, signal levels were extracted from their background by applying spot edge detection methodology [7]. This method locates the average intensity around the spot's outline and then applies this as the background for the spot. The background was, therefore, calculated independently for each spot, and signal levels (normalized to mean counts) were calculated independently for each spot (ImageQuant software; Amersham, Piscataway, NJ, USA). Low-quality spots were also filtered for quality control, and background noise was eliminated from the analysis. Universal probes were placed on two lanes on each membrane to serve as standards, and signal levels were converted to mean counts by comparison with standards on the membrane. Signal levels were then adjusted for abundance by comparing them to the universal control probes. This approach allowed for computing the abundance of the target species individually by adjusting the DNA concentration in each sample.

### 2.5. Saliva Sampling

Paraffin-stimulated whole saliva samples were collected between 9 and 12 a.m. from children who had refrained from eating and drinking for 2 h. The saliva collection was performed with the children seated, head tilted slightly forward, and eyes opened for a period of 2 minutes. Samples were collected on ice, and they were immediately centrifuged at 13000 rpm for 5 minutes to remove insoluble material, and all procedures were performed at 4°C. The supernatant was removed and placed in eppendorf tubes that were stored at −80°C.

### 2.6. MS Proteomic Analysis of Saliva Samples

Cy dyes were purchased from Amersham Pharmacia Biotech (Picataway, NJ, USA). Thawed saliva samples were processed at 4°C. Two types of chips with different surface affinity were used in the protocols. CM10 and Q10 anion exchange ProteinChip (Ciphergen Biosystems Inc., Fremont, Calif, USA) surfaces were equilibrated with 150 *μ*L of binding buffer (100 mM Tris-Hcl, pH 9.0). Individual saliva samples were mixed with denaturing buffer (9 M urea and 2% CHAPS), at a ratio of 2 : 3. Each of the denatured samples (10 *μ*L of each) was applied in duplicate with 90 *μ*L of binding buffer to the pre-equilibrated chips. ProteinChip arrays were incubated for 60 min at room temperature with vigorous shaking, washed twice with binding buffer for 5 min each, followed by two washes with distilled water. Arrays were dried at room temperature for 15 min followed by two additions (1 *μ*L each) of a 50% solution of sinapinic acid (Sigma) prepared in 50% acetonitrile and 0.5% trifluoroacetic acid (TFA). Sample handling, including deposition of matrix, was performed on a Biomek 2000 automated work station (Beckman-Coulter, Thousand Oaks, Calif, USA) using two 96-well Bioprocessors (Ciphergen). Samples were analysed using SELDI-TOF-MS (Protein Biology System II, Ciphergen Biosystems). Each chip was shot twice with different (low and high) laser intensity. All spectra consisted of 130 averaged laser shots and were externally calibrated using All-in-One Protein Standard II (Ciphergen Biosystems), containing seven calibrants between 7 and 147 kDa. Spectral data were processed similarly using Ciphergen Express 3.1 data management software. The whole saliva proteome data consisted of 2 groups: caries-active children (*n* = 86) and caries-free children (*n* = 118). Equivalent numbers of children in each group were the same for chip type and laser intensity.

### 2.7. MS Data Preprocessing

The MS profile preprocessing and interpretive analysis was performed using proteomic data analysis package (PDAP) developed at the University of Pittsburgh [22] and implemented in MATLAB (MathWorks Inc.) PDAP supports all steps of SELDI-TOF-MS data analysis including profile preprocessing, peak selection, univariate and multivariate feature selection methods, classification, evaluation, and validation methods. We applied five preprocessing steps implemented in the PDAP: (1) variance stabilization, (2) baseline correction, (3) intensity normalization, (4) smoothing, and (5) profile alignment steps [22]. Briefly, we applied the following PDAP preprocessing choices: cube-root variance stabilization, PDAP's baseline subtraction routine based on the local moving window of width 200 time-points, total ion current normalization restricted to the range of 1500–16500 Daltons, Gaussian-kernel smoothing, and the peak-based dynamic programming alignment. None of the profiles used in the study exhibited total ion current (TIC) value that differed by more than two standard deviations from the mean TIC, which is our current quality-assurance/quality-control threshold for sample exclusion. Following preprocessing, replicate spectra for each patient were averaged to create a single mean profile per patient.

### 2.8. MS Peak Selection

The majority of proteomic data analyses in the literature restrict their attention only to information represented in the peaks of the signal. To perform peak selection, we applied a two-stage procedure implemented in PDAP [22]. The procedure first identifies all peak positions; afterwards, it assigns intensities to such positions in each profile. The *peak identification stage* works with the mean profile obtained by averaging all profiles in the training data. The approach is robust enough even if a specific peak is not recorded in all profiles, whilst it tends to average out random signal fluctuations. The peak detection procedure relies on a local max window approach in which the position is considered to be a peak only if it is maximal with respect to signals in its close local neighborhood. To *assign intensity value to every peak* in a profile, we use the average of readings in a local neighborhood of the peak location. Such a method reduces the chance of a noisy reading at a single *m*/*z* position. Through these techniques, we reduce every child's spectrum to a list of peak positions and their intensities. Peaks within the range of 1,500 and 40,000 Da are considered. This lets us concentrate on a less noisy, more meaningful portion of the mass spectrum.

### 2.9. Statistical Analysis of Data

The microbial and proteomics data were analyzed using both univariate and multivariate statistical methods implemented in the proteomic data analysis package (PDAP) [22]. The classification approach was used to determine if differences in bacterial levels were present between caries-free and caries-active subjects or in search of diagnostic markers for early detection of caries disease in saliva. The analyses were first performed separately for each data type. After that proteomic and genomic data were analyzed in combination.

### 2.10. Univariate Analysis

The objective of the univariate analysis is to identify features (microbial species or MS peaks) that can discriminate between case and control (caries-active and caries-free) profiles. A number of univariate scores that allowed comparing relatively each of the potential biomarkers exist. Those include correlation, Fisher, *t*-statistics, or chi-square score as well as scores derived from *P*  values of statistical tests. We use a score based on the Wilcoxon rank-sum test in our analysis.

### 2.11. Multivariate Analysis

The objective of multivariate analysis is to build a predictive model *f* : **X** → *Y* that can, with a high accuracy, assign correct class labels Y (case or control) to patients' measurements (**X**). In contrast to the univariate analysis all profiles features and their combinations are considered. We adopt a machine-learning approach in which a model is learned and evaluated from the data in the study. 

The quality of each classification model is verified by using random resampling validation schemes [23, 24]. Briefly, the goal is to evaluate the generalization performance of the prediction model, that is, its performance on samples we expect to see in the future. Since these are not available, we split the data available to us into the training and test set. The model is always learned on the training set and tested on the test set. The split of 70 : 30 is used to divide the data into training and testing sets. Once the model is developed on the learning set, it is never modified again. To reduce the chance of a possible bias due to a lucky or unlucky split, the random subsampling, an approach with 40 different splits [23] is applied to evaluate the predictive performance of the model. The average statistics reported include test errors, sensitivity, and specificity of the model.

A number of different classification models and algorithms suitable for the learning task exist. In this work, we report the results of two classification models: the linear support vector machine (SVM) [25–28]. All these methods are quite robust when applied to high-dimensional data. In addition, we test the proteomic data also on the SVM model with apriori feature selection via feature filtering based on the *P* value of the Wilcoxon rank-sum test.

## 3. Results

### 3.1. Study Population

A total of 204 children with an average age of 3.83 ± 2.55 years received an oral examination and were sampled for microbial plaque and saliva. Parents/guardians reported that most of the children had never been seen by a dentist (>96%), and those that had seen a dentist were seen for emergency care only. Based on clinical examination, 118 children (60 females, 58 females, mean age 2.3 ± 0.2 years) were determined to be caries-free (caries-free group) with a surface-based caries prevalence rate (SBCPR) = 0), and 86 children (40 females, 46 males, mean age 6.02 ± 0.2 years) were determined to have caries (caries-active group); none of the group had existing restorations (with a mean SBCPR = 17.23% ± 10.70%).

### 3.2. Analysis of Microbial Data

**Figure 1:**
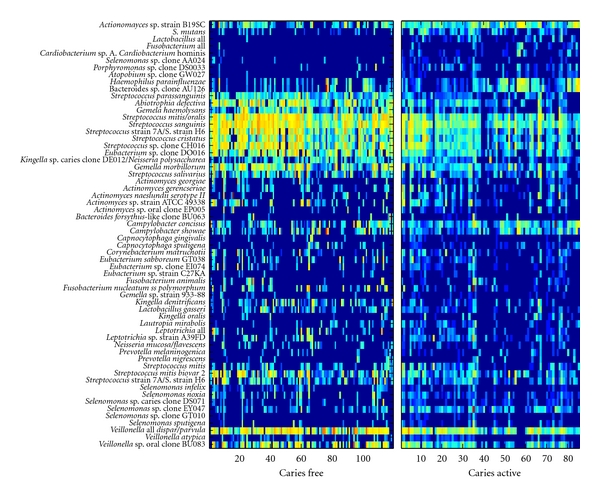
Expression levels for bacterial species measured by multiplexed array technology. The left panel displays bacteria abundance levels for all caries-free patients, while the right panel displays bacteria abundance levels for caries-active patients. Each column of the panel represents the measured bacterial abundance levels of a single patient. Each row is marked on the right with its corresponding bacterial species or group. The color bar to the left indicates the level of abundance. Brighter colors indicate higher abundance level of the particular bacteria measured. Indicators of caries exhibit differential expression in the caries-active versus caries-free group. Note that the top 10 rows do not show much expression in the caries-free group yet are noticeably expressed in the caries-active group.

Figure 1 shows the expression levels (abundance) of bacterial species or groups for caries-free and caries-active samples. We see that increased abundance levels of bacterial species or groups on the left and the suppression of abundance levels of bacterial species or groups on the right indicates the occurrence of disease (dental caries). Intuitively, these correspond to communities of beneficial and detrimental bacteria. Notably, species such as *S. mutans* and lactobacilli that are often associated with dental caries are less abundant in caries-free children relative to caries-active children, whereas a number of beneficial species or of species that are not associated with dental caries such as *Streptococcus mitis/oralis*, *Streptococcus sanguinis*, and *Streptococcus cristatus* are more abundant in caries-free children relative to caries-active children. 

**Figure 2:**
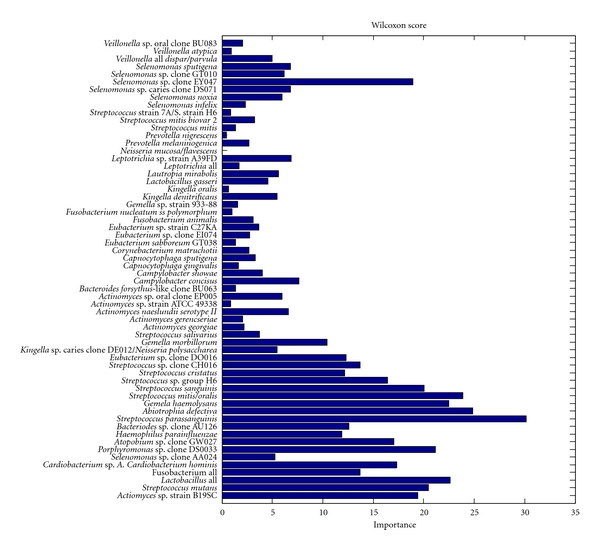
Importance of bacteria probes according to their individual discriminative power. Species of bacterial species or group are indicated along the *y*-axis. Shaded bars indicate the importance of the species as measured by the Wilcoxon rank-sum score (the score is calculated as −log⁡*P*, where *P* is the *P* value of the test). A larger importance indicates a larger propensity for the levels of that bacterial specie or group to be differentially expressed in the caries-free versus the caries-active group. *S. parasanguinis* appears to be the most differentially expressed bacterial marker of caries, followed by *Abiotrophia defectiva. *

Figure 2 illustrates the distribution of the univariate scores based on the Wilcoxon rank-sum test for the bacterial array probes in the study. The top 10 bacterial species or groups according to the score which that have been definitely and/or that could be possibly implicated in caries onset and progression included *S. parasanguinis, A. defectiva, S. mitis/oralis, G. haemolysans, S. mutans*, lactobacilli, *Actinomyces* sp. strain B19SC, Selemonas sp. clone EY047, *Atobopium* sp. clone GW027, and *Porphyromonas* sp. clone DS033.

### 3.3. Multivariate Classification Analysis

**Figure 3:**
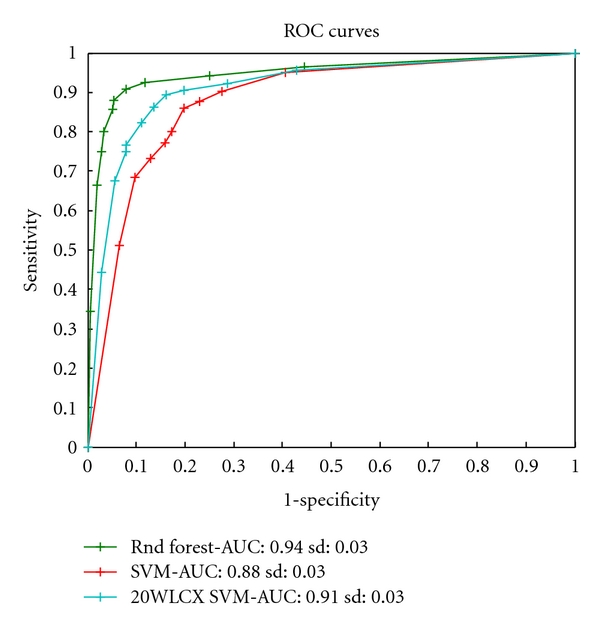
Receiver operating characteristic (ROC) curves for three classification methods built for the microbial data. ROC curves reflect the tradeoffs in between sensitivity and specificity for caries-active detection. A higher curve generally indicates a better method. The AUC (area under the curve) statistic summarizes the tradeoffs across varied sensitivity/specificity range. The random forest model appears to be the best classification model for microbial data.

Multivariate analysis exploring the predictive performance of three multivariate classification models shows that the test error classification performance varied between 8.4%–15.65%, sensitivity between 82% and 87.5% and specificity between 86.24% and 94.91% (Table 1). These results were obtained by optimizing the misclassification error. In addition to the classification analysis in Table 1, we have also varied the costs of misclassifications to obtain the ROC of the methods and their area under the receiver-operator-characteristic (ROC) (area under the ROC curve = AUC) statistic (Figure 3). Out of the three models tested the random forest classifier achieved the best performance. 

**Figure 4:**
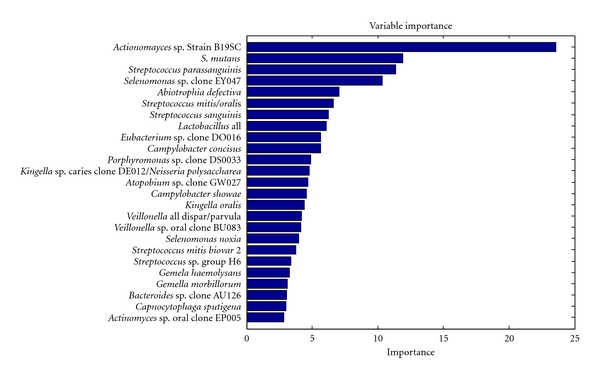
Relative importance of bacterial DNA probes for classifying caries-active and caries-free samples using the random forest model. The 25 most significant DNA probes are listed, and the shaded bars display their importance. The five most important probes are *Actinomyces* strain B19SC, *S. mutans*, *Streptococcus parasanguinis*, *Selenomonas* sp. Clone EY047, and *Abiotrophia defectiva*.

Figure 4 shows the importance of species for the performance of the random forest classifier using the relative importance measure offered by the method. The top 25 species and their scores are shown. Unlike univariate scores (see Figure 2), the multivariate scores assess the importance of the feature in context of other features in the panel. The differences among scores can be explained by correlations that exist among species and their “substitutability”. In such a case, the relative importance of two highly correlated biomarkers in the multivariate panel may be decreased. Although there is some overlap, bacteria that were important in classifying caries-active (such as *Actinomyces* strain B19SC, *S. mutans*, and Lactobacilli all) and caries-free groups (such as *S*. *parasanguinis*, *Abiotrophia defective*, and *S. mitis/oralis*) using the random forest model are different than those identified with the Wilcoxon rank-sum score (Figure 2), suggesting that there may be critical changes in the caries and health associated biofilm microflora, and quantitative changes (expressed as abundance) in specific bacteria may serve as biomarkers.

### 3.4. Analysis of Proteomic Data

**Figure 5:**
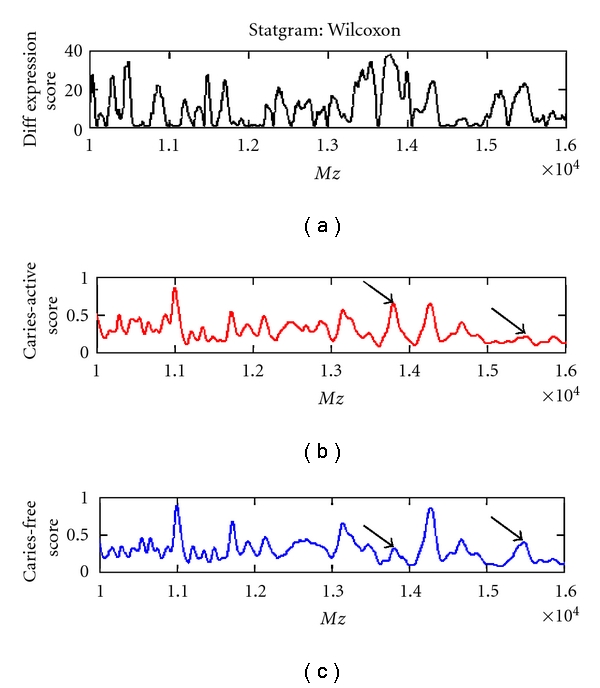
Statgram for the Wilcoxon rank-sum score measuring the expression differences between proteomic profiles for caries-active and caries-free groups. (a): the Wilcoxon rank-sum score is plotted for each feature in the proteomic profile. Higher score values indicate a larger differential expression between caries-active and caries-free profiles. (b): a plot of the mean proteomic profile for the caries-active group. (c): a plot of the mean proteomic profile for the caries-free group. Two peaks in the mean profiles are marked with arrows. The difference in peak height at the arrows suggests differential expression and is confirmed by a higher value in the Wilcoxon score for those peaks.

**Figure 6:**
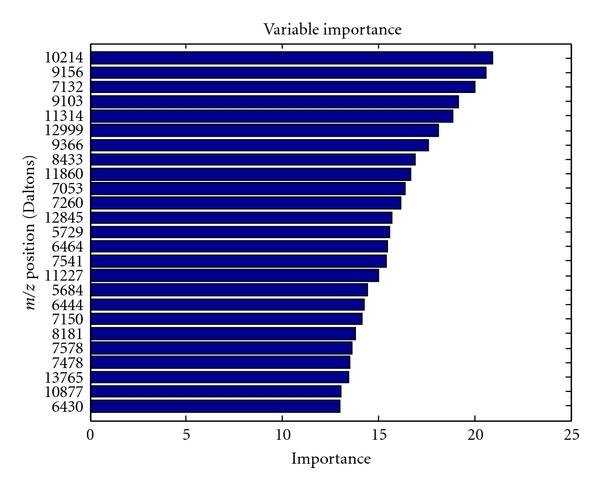
Importance of proteomic profile peaks according to their individual discriminative power. Mass-to-charge (*m*/*z*) positions of discriminative peaks are indicated along the *y*-axis. Shaded bars indicate the importance of the peak as measured by the Wilcoxon rank-sum score. A larger importance indicates a larger propensity for that particular profile peak to be differentially expressed in the caries-free versus the caries-active group. The most differentially expressed profile peak appears at 10214 Daltons, followed by the peak at 9156 Daltons.

As a first step of the analysis, we have studied MS profiles using univariate statistical methods. Briefly, each profile species was judged by its ability to discriminate caries-active and caries-free samples. Similarly to the microbial data, a nonparametric Wilcoxon rank-sum test was applied.  Figure 5 illustrates this on a statgram for the Wilcoxon rank-sum test after it was applied to the MS profiles for CM-10 chips. The view is restricted to the range of 11,000–16,000 Daltons. Mean profiles for case and control groups are also observed. The Wilcoxon rank-sum score is greatest for features, which exhibit a large difference between intensities of the mean group profiles.  Figure 6 shows the score for the top 25 Wilcoxon peaks.

### 3.5. Multivariate Classification Analysis

Table 2 displays the performance statistics obtained by three predictive models: SVM, SVM on the top 100 Wilcoxon peaks, random forest; on four different datasets obtained for two different chips: CM-10 and Q-10, each shot with two laser intensities: high and low. The results represent average test error, sensitivity, and specificity. Test errors are in the range of 22.73% to 35.68%, which is much better than the expected error under a fully random classifier, 45.6%. Sensitivity ranges from 54.24% to 75.82%, while specificity ranges from 69.80% to 83.20%. 

**Figure 7:**
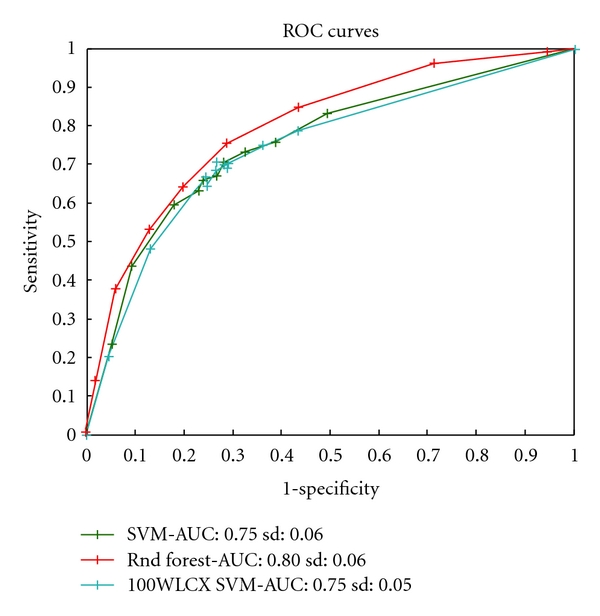
Receiver operating characteristic (ROC) curves for three classification methods built for the CM-10 low-intensity proteomic data. ROC curves reflect the tradeoffs in between sensitivity and specificity for caries-active detection. A higher curve generally indicates a better method. The AUC (area under the curve) statistic summarizes the tradeoffs across varied sensitivity/specificity range. Standard deviations (sd) of the statistic are also reported. The random forest model appears to be the most effective classification method.

Out of the four types of spectra analyzed the two that seem to perform best are spectra obtained for low laser intensity settings. We suspect this is caused by an increased chance of fragmentation of species for high-intensity settings. Figure 7 shows the results of the full ROC analysis for one of the low-intensity datasets, CM-10 low. The random forest model appears to be the most effective classification method.

The results of classification analyses reveal that it is definitely possible to observe a discriminative pattern in the proteomic spectra. However, the signal appears weaker than the signal found in the microbial data. This can be explained by the fact that SELDI-TOF-MS detects more reliably and reproducibly protein species that are more abundant in the saliva specimen. It is quite possible that some highly discriminative proteins for caries-active and caries-free groups occur in saliva at lower concentrations, and thus are not detected due to the inherent limits of the SELDI-TOF-MS profiling technology. 

**Figure 8:**
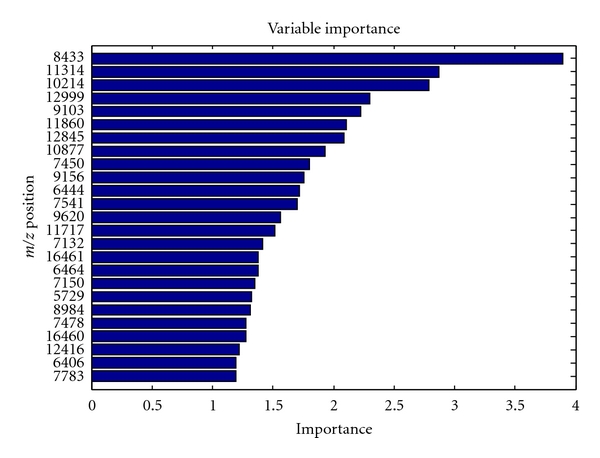
Relative importance of proteomic profile peaks for classifying caries-active and caries-free samples using the random forest model. The mass-to-charge (*m*/*z*) positions of 25 most important peaks are listed (*y* axis), and the shaded bars display their importance. *m*/*z* positions are given in Daltons. Note that the relative importance of the peak position for multivariate classifier may differ from its individual (univariate) importance (see Figure 6).

Figure 8 shows the relative importance of peaks for the classification accuracy of the random forest model for the CM-10 low dataset. Only the top 25 peaks are shown. Features with high importance are interpreted as being very relevant for the classification task and that they cooperate well with other features in the panels. Once again, note the differences in between peak species in Figures 8 and 6: Wilcoxon rank-sum score evaluates every peak independently, while multivariate methods such as random forest aim to evaluate each peak feature in combination with other peak features.

### 3.6. Analysis of Combined Microbial and Proteomic Data

Lastly, in order to determine whether or not the microbial and proteomic data contained collaborative information, we matched the patients and appended the microbial features to the list of peak features in the proteomic CM-10 low data.

### 3.7. Multivariate Classification Analysis

Table 3 shows the classification statistics obtained by three classification models: SVM, SVM on the top 100 Wilcoxon features, and random forest. After merging the two datasets, test errors results range from 6.00% to 16.05%, sensitivity from 76.52% to 92.68%, and specificity from 91.14% to 95.20%. Particularly good are results for the SVM model restricted to the top 100 Wilcoxon features that yields 6% average test error. 

The comparison of results for the combined and independent data suggests that the combination of MS proteomic and microbial sources is beneficial for the classification accuracy and that combined data lead to improved predictive models for caries-active and caries-free patients. In particular, the linear SVM classifier errors fell from 16% on the microbial data and 26% on the MS proteomic data to approximately 9% on the combined data. Similarly, a feature restricted linear SVM (with Wilcoxon feature filtering) improved from 11% on the microbial data and 26% on the proteomic data to 6% error on the combined data. The only classifier that did not yield an improvement on the combined data was random forest. The method achieved 9% test errors on the microbial data and 32% on the MS proteomic data, while the combination resulted in 16% test errors. We conjecture the drop (from low test errors on microbial data to higher errors on proteomic and combined data) is the effect of higher-dimensional data on the classification accuracy of the random forest model: the microbial dataset includes 60 features while the MS proteomic dataset includes about 2000 peaks. In contrast to this, the performance of the SVM classifier appears more robust in the presence of high dimensional data. To verify this conjecture we run the random forest classifier on the top 100 Wilcoxon features and obtained average test error of 8.68%, at 87.78% sensitivity and 94.45% specificity, which appears to support our conjecture.

**Figure 9:**
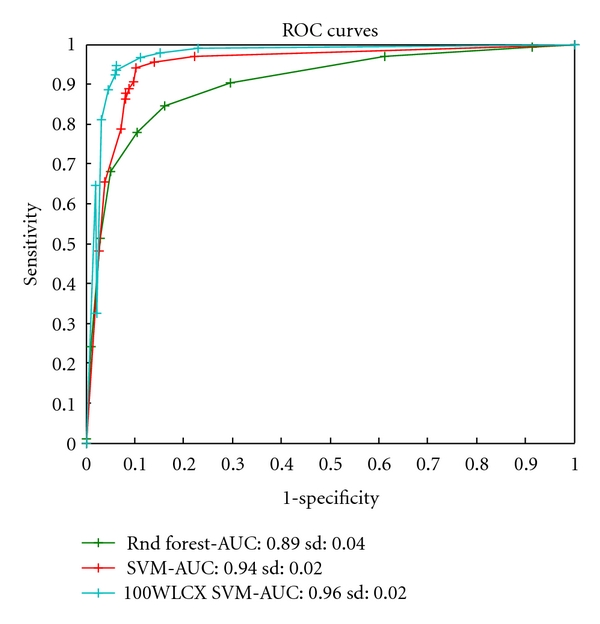
Receiver operating characteristic (ROC) curves for three classification methods built for the CM-10 low-intensity proteomic and microbial combined data. ROC curves reflect the tradeoffs in between sensitivity and specificity for caries-active detection. A higher curve generally indicates a better method. The AUC (area under the curve) statistic summarizes the tradeoffs across varied sensitivity/specificity range. Standard deviations (sd) of the statistic are also reported. The SVM based on only the top 100 Wilcoxon-scored features appears to be the best method.

Results of similar nature to Table 3 are obtained if we perform full ROC analysis of the three methods (Figure 9) and calculate the area under the ROC curve (AUC) statistic. The area under the curve suggests that the combined data improve the ability of the SVM model to classify correctly case and control samples under varied preferences on different types of misclassification errors.

## 4. Discussion

The current study was performed in a cohort of young children likely to represent a natural history of dental caries development in an at-risk population. While previous epidemiologic and laboratory studies indicate that oral microbes and components of the salivary proteome are risk factors for development of caries [1–4, 9, 10], the use of high-throughput methodologies to characterize the bacterial biofilm and the salivary proteome permit further large-scale clinical sampling and testing to validate earlier studies. However, the oral flora and the salivary proteome are both complex and neither is static. This study represents an application of a statistical machine learning principle to predictive model construction. We used relatively high-throughput methodologies to characterize the oral flora and the salivary proteome, multiplexed microbial arrays, and SELDI-TOF-MS profiling. Using this approach, we have demonstrated experimentally that the data obtained by these two technologies carry information useful for discriminating caries-active and caries-free patients with high accuracy. Our results show that microbial data are more powerful for classification purposes than MS proteomic data, if the two data sources are analyzed independently. However, the two data sources also appear to carry nonoverlapping information that leads, when they are combined, to improved classification performance and improved discrimination of caries-free and caries-active patients. Analysis of combined datasets resulted in reduced test error and improved sensitivity and specificity (Table 3, Figure 9), indicating that data from these different sources may ultimately permit identification of more clinically useful biomarkers for disease. 

The advent of molecular genetic methodologies to characterize the oral flora in health and disease is revealing the complexity of oral biofilms [29]. Use of 16S DNA profiling is employed to establish the flora associated with different sites in the oral cavity and indicating that the flora of specific niches differs between health and disease. Recent studies characterizing the dental flora in caries-free and caries-afflicted individuals suggests that the microbial flora associated with dental caries is more complex than originally thought and quantitative shifts in the relative amounts of multiple oral microbes can be linked to the development of dental caries [6–8]. Given the biofilm concept of oral flora associated with tooth surfaces, these findings are not unexpected. A corollary of this microbial scenario is that identification of key microbes that are not only present but also quantitatively altered in health and disease states may contribute to the development of a clinically useful set of biomarkers. The current study quantitated relative levels of 82 bacteria sampled from tooth surfaces in health and disease and developed a model to distinguish cases and controls with sensitivity and specificity of 86% and 90%, respectively (Table 1). 

Dental caries is a chronic process, which also demonstrates a bidirectional quality early in the disease process. In the current study population, of the 118 caries-free individuals, 10 individuals showed the caries-associated flora shift previously identified [7]. Our classification model predicted that the microbial profile of these individuals were similar to the caries-active group even though clinically they showed no signs of disease. In a subsequent follow-up clinical examination of the study population one year later, we found that all 10 individuals manifested clinically evident caries. These findings suggest that the change in the oral flora previously associated with clinical caries preceded the clinical appearance of disease. Results obtained for the modeling of microbial expression data in the current study, therefore, may be regarded as fairly good, given that some individuals may appear clinically as healthy, but demonstrate the flora associated with disease.

Quantitative and qualitative aspects of saliva have long been proposed as etiologic factors in dental caries [2]. Evidence that decreased salivary flow is positively associated with increased dental caries is substantial, and correlations are found with natural disease states, such a Sjogren's syndrome as well as iatrogenic induced states such as following radiation treatment that ablate salivary glands [2]. Evidence for a role for specific salivary proteins as contributory or protective in the caries process is less certain [11, 30]. Part of the difficulty may be related to the fact that the great abundance of certain salivary proteins makes it difficult to identify changes in levels of proteins that are present at much lower amounts. SELDI-TOF-MS offers a simple yet high-throughput and very sensitive proteomic approach that allows protein expression profiling of large sets of complex biological specimens [13]. Importantly, this approach permits assessment of low mass proteins (<10 kDa), which are difficult to effectively assay by other means. While SELDI-TOF-MS does permit evaluation of a potentially broad range of proteins, it has certain limitations, including the inability to identify specific proteins. SELDI-TOF-MS has been used to successfully detect salivary biomarkers [31, 32]. Evaluation of SELDI-TOF-MS protein peaks to distinguish cases and controls resulted in a fair model, but one that was inferior to the microbial dataset alone. We believe the model may be improved with the ability to detect and identify specific proteins including those present in smaller amounts in saliva. Efforts are currently underway to characterize the salivary proteome and should permit identification and quantification of salivary proteins in a high-throughput fashion [17, 19, 33]. 

A goal of the study was to determine if data from both microbial and proteomic sources could both improve the predictability and sensitivity and reduce the error compared to individual microbial or proteomic models. Our current findings suggest that this is, in fact, the case (Table 3). These findings are consistent with an etiologic role for oral microbes and salivary proteins but also suggest that some of these factors are independent of each other. These findings suggest that characterization of both the microbial and salivary proteome may provide better predictive value for identification of individuals at risk for developing childhood caries. Identification of these microbial and proteomic variables may also permit a more refined understanding of the underlying disease process and clarify significant etiologic factors important in the shift from health and disease. Such data may permit identification of individuals who have not developed clinical disease but who manifest the microbial and salivary biomarker signature that suggests that they are at risk to develop the disease. This will permit intervention in a presymptomatic state. This is particularly important to early childhood dental caries, as the disease is believed to be reversible in its early stage [34]. Further refinement of clinically useful microbial and salivary biomarkers will aid risk assessment and identification of therapeutic targets. In addition, such biomarker profiling may provide therapeutic endpoints, permitting determination of successful treatment to modify microbial and proteomic profiles correlated with dental caries susceptibility.

## 5. Conclusions

We have demonstrated the use of relatively high-throughput methodologies to characterize the oral flora and the salivary proteome in young children at risk for childhood caries. Using a statistical machine learning approach, we have demonstrated experimentally that the data obtained by these two technologies carry information useful for discriminating caries-active and caries-free patients with high accuracy. Our results show that microbial data are more powerful for classification purposes than MS proteomic data, if the two data sources are analyzed independently. However, the two data sources also appear to carry nonoverlapping information that leads, when they are combined, to improved classification performance and improved discriminability of caries free and caries active patients. Analysis of combined datasets resulted in reduced test error and improved sensitivity and specificity, indicating that data from these different sources may ultimately permit identification of more clinically useful biomarkers for disease. 

Identification of these microbial and proteomic variables may ultimately permit a more refined understanding of the underlying disease process and clarify significant etiologic factors important in the shift from health and disease. Such data may permit identification of individuals who have not developed clinical disease but who manifest the microbial and salivary biomarker signature that suggests that they are at risk to develop the disease. This will permit intervention in a presymptomatic state. This is particularly important to early childhood dental caries as the disease is believed to be reversible in its early stage. Further refinement of clinically useful microbial and salivary biomarkers will aid risk assessment and identification of therapeutic targets. In addition, such biomarker profiling may provide therapeutic endpoints, permitting determination of successful treatment to modify microbial and proteomic profiles correlated with dental caries susceptibility.

## Figures and Tables

**Table 1:** Performance statistics of three (multivariate) classification models built for the microbial data. The models were optimized for the average misclassification error (zero-one loss). The statistics include averages and standard deviations of test errors, sensitivities, and specificities of respective classifiers. The averages and standard deviations were calculated across 40 different train/test obtained using the random subsampling approach.

Classifier	Test error	Sensitivity	Specificity
“SVM”	15.65% ±3.87%	81.98% ±6.65%	86.24% ±5.12%
SVM 20 WLCX	11.77% ±23.674%	86.05% ±7.36%	90.11% ±4.59%
“RF”	8.31% ±3.15%	87.51% ±6.51%	94.91% ±3.23%

SVM: linear support vector machine.

SVM on the top 20 Wilcoxon peaks.

Random forest.

**Table 2:** Performance statistics of three classification models tested on the MS proteomics data. The models were optimized for the average misclassification error (zero-one loss). Four different MS datasets generated for combinations of two affinity chips (CM-10 and Q-10) and two intensity instrument settings (high and low) were analyzed. The statistics include averages and standard deviations of test errors, sensitivities, and specificities of respective classifiers. The averages and standard deviations were calculated across 40 different train/test obtained through the random subsampling approach.

“caries cm 10 high”	Test error	Sensitivity	Specificity
SVM	31.82% ±5.35%	66.10% ±7.49%	69.80% ±9.44%
SVM 100 WLCX	35.68% ±5.58%	57.72% ±21.56%	70.93% ±18.72%
Rnd Forest	32.95% ±5.74%	54.24% ±12.29%	79.17% ±10.84%

“caries cm10 low”	Test error	Sensitivity	Specificity

SVM	28.23% ±5.82%	69.83% ±8.19%	73.81% ±9.62%
SVM 100 WLCX	26.68% ±5.78%	73.64% ±11.66%	73.55% ±10.66%
Rnd Forest	25.64% ±5.76%	65.29% ±9.55%	83.20% ±9.54%

“caries q10 high”	Test error	Sensitivity	Specificity

SVM	25.91% ±4.88%	73.31% ±6.87%	75.05% ±8.14%
SVM 100 WLCX	25.91% ±4.88%	73.31% ±6.87%	75.05% ±8.14%
Rnd Forest	32.00% ±4.47%	57.41% ±11.21%	78.31% ±9.70%

“caries q10 low”	Test error	Sensitivity	Specificity

SVM	22.73% ±3.93%	75.82% ±9.62%	78.88% ±6.94%
SVM 100 WLCX	26.14% ±4.85%	71.91% ±13.00%	75.45% ±9.07%
Rnd Forest	25.50% ±5.64%	69.39% ±11.23%	79.99% ±9.34%

SVM: linear support vector machine.

SVM on the top 100 Wilcoxon peaks.

Random forest.

**Table 3:** Performance statistics of three classification models tested on the combined microbial and MS proteomics data. The models were optimized for the average misclassification error (zero-one loss). For this experiment, only spectra for CM-10 low dataset were used and combined with the microbial data. The statistics include averages and standard deviations of test errors, sensitivities, and specificities of respective classifiers. The averages and standard deviations were calculated across 40 different train/test obtained through the random subsampling approach.

Classifier	Test error	Sensitivity	Specificity
“SVM”	8.91% ±3.42%	89.61% ±5.76%	92.36% ±4.55%
SVM 100 WLCX	6.00% ±2.67%	92.68% ±4.46%	95.20% ±3.87%
“RF”	16.05% ±6.26%	76.52% ±9.63%	91.14% ±7.76%

SVM: linear support vector machine.

SVM on the top 20 Wilcoxon peaks.

Random forest.
